# Type IV secretion system of *Brucella* spp. and its effectors

**DOI:** 10.3389/fcimb.2015.00072

**Published:** 2015-10-13

**Authors:** Yuehua Ke, Yufei Wang, Wengfeng Li, Zeliang Chen

**Affiliations:** ^1^Institute of Disease Control and Prevention, AMMSBeijing, China; ^2^Department of Laboratory Medicine, General Hospital of Chinese People's Armed Police ForcesBeijing, China; ^3^Department of Orthopedics, The First Affiliated Hospital of General Hospital of People's Liberation ArmyBeijing, China

**Keywords:** *Brucella*, type IV secretion, intracellular survival, effector, signaling pathway

## Abstract

*Brucella* spp. are intracellular bacterial pathogens that cause infection in domestic and wild animals. They are often used as model organisms to study intracellular bacterial infections. *Brucella* VirB T4SS is a key virulence factor that plays important roles in mediating intracellular survival and manipulating host immune response to infection. In this review, we discuss the roles of *Brucella* VirB T4SS and 15 effectors that are proposed to be crucial for *Brucella* pathogenesis. VirB T4SS regulates the inflammation response and manipulates vesicle trafficking inside host cells. VirB T4SS also plays crucial roles in the inhibition of the host immune response and intracellular survival during infection. Here, we list the key molecular events in the intracellular life cycle of *Brucella* that are potentially targeted by the VirB T4SS effectors. Elucidating the functions of these effectors will help clarify the molecular role of T4SS during infection. Furthermore, studying the effectors secreted by *Brucella* spp. might provide insights into the mechanisms used by the bacteria to hijack the host signaling pathways and aid in the development of better vaccines and therapies against brucellosis.

## Introduction

*Brucella* spp. cause brucellosis in domestic and wild animals. Their primary hosts include sheep, cattle, swine, dogs, camels, and desert woodrats (Martirosyan et al., 2011; Bargen et al., 2012). Humans are secondary or accidental hosts and usually contract brucellosis through contact with infected animals or animal products (Atluri et al., 2011; Bargen et al., 2012). Brucellosis is a chronic disease and may last several weeks or months. If it is not treated effectively, the disease can lead to pathologies in the liver, spleen, lymph nodes, bone marrow, reproductive tract, and skeletal system (Atluri et al., 2011). Some complications, including arthritis, liver abscess, epididymo-orchitis, neurobrucellosis, and endocarditis, often tend to relapse, resulting in long-term disability and suffering (Colmenero et al., 1996; Franco et al., 2007).

In natural hosts, *Brucella* can penetrate the mucous membranes of the reproductive, respiratory, and digestive tracts and the conjunctiva; in humans, the most common sites of entry are the respiratory and digestive tracts (Atluri et al., 2011; Bargen et al., 2012). Once the bacteria break the mucosal barrier, they enter the regional lymph nodes and disseminate through the body (Atluri et al., 2011). Inside the host, the bacteria reside in the macrophages for several days and replicate to high numbers asymptomatically (Martirosyan et al., 2011). Therefore, elucidating the mechanisms involved in their intracellular survival and their ability to evade host immunity is crucial for understanding the pathogenesis of *Brucella* spp., which are used as model organisms to study intracellular bacterial infections.

On entering the host cells, the bacteria interact with the early and late endosomes and acquire several markers, including Rab5, early endosome antigen (EEA) 1, and Rab7, resulting in the formation of a “*Brucella*-containing vacuole” (BCV) (Pizarro-Cerdá et al., 1998; Chaves-Olarte et al., 2002; Celli et al., 2003; Starr et al., 2008; Lee et al., 2013). The BCVs then fuse rapidly with the lysosome in a controlled manner, as suggested by the presence of the lysosomal markers, lysosomal-associated membrane protein (LAMP), and CD63, but not the luminal lysosome enzyme, cathepsin D, on the surface of bacteria (Pizarro-Cerdá et al., 1998; Celli et al., 2003; Starr et al., 2008). In this transient stage, most of the contents of the BCVs are subjected to phagolysosomal degradation, and 90% of internalized *Brucella* are killed by the action of hydrolyzing enzymes (Celli et al., 2003). However, the remaining 10% evade the host killing mechanisms through an unknown mechanism that probably involves the acidification of the BCVs, subsequent triggering of the *virB* operon [which encodes the type IV secretion system (T4SS)], and release of a large variety of effectors into the host cells' cytosol (Boschiroli et al., 2002). The bacteria then traffic and arrive at the endoplasmic reticulum (ER). Within the ER, the bacteria survive and establish their replicative niche, and multiply to large numbers (Celli et al., 2003). However, the ER is not the final site of this intracellular journey; recent studies have shown that autophagy-like vacuoles provide a replication-permissive compartment for *Brucella* following the ER stage (Starr et al., 2012). Because the “*Brucella*-replicating organelle” possesses autophagic features, it is also called the autophagic BCV (aBCV). The formation of the aBCV is essential for the completion of the intracellular lifecycle of *Brucella* and for cell-to-cell spreading (Starr et al., 2012).

Although several studies have attempted to elucidate the pathogenesis of *Brucella*, the components that are responsible for their virulence and their ability to establish systemic infection have not been identified. For a long time, it was thought that *Brucella* spp. lacked the characteristic virulence factors that are present in other bacteria (Fugier et al., 2007). However, in recent years, various virulence factors that are essential for infection, including lipopolysaccharide (LPS) (Lapaque et al., 2005), β-cyclic glucan (Arellano-Reynoso et al., 2005; Martirosyan et al., 2012), BvrS/BvrR (Guzman-Verri et al., 2002; Martín-Martín et al., 2012), outer membrane proteins (Omps) (Lim et al., 2012; Vizcaíno and Cloeckaert, 2012), BmaC (Posadas et al., 2012), MucR (Mirabella et al., 2013), SagA (Del Giudice et al., 2013), BtaE (Ruiz-Ranwez et al., 2013), BacA (Martín-Martín et al., 2012), and BetB (Lee et al., 2014), have been identified in *Brucella* VirB. T4SS is another key virulence factor that plays important roles in mediating intracellular survival and manipulating host immune response to infection. Many studies have elucidated various aspects of VirB T4SS from *Brucella* spp., such as its genetic organization, structure, functions, and transcriptional regulation. Some studies have used T4SS as targets for small molecular inhibitors (Paschos et al., 2011; Smith et al., 2012). T4SS functions in the translocation of effector proteins across bacterial and host membranes into the host cytosol. Therefore, to understand the functions of T4SS, it is important to identify these effectors. Recent studies have identified 15 effectors that are secreted by *Brucella* in a T4SS-dependent manner. In this review, we discuss the roles of *Brucella* T4SS and its 15 effectors, as well as the associations between bacterial phenotype and effector functions.

## Brucella type IV secretion system

### Organization, structure, and regulation

In *Brucella*, T4SS is encoded by the *virB* operon, which consists of 12 genes (*virB1–12*) located on chromosome II. Transcription of the *virB* operon is controlled by the promoter upstream of *virB1* (O'Callaghan et al., 1999; Sieira et al., 2000). The *virB* operon was first identified in *B. suis*. Subsequently, it was found to be highly conserved in all *Brucella* spp. for which genomic sequences were available, indicating a potentially critical role for this operon (O'Callaghan et al., 1999). Although the structure of T4SS from *Brucella* is unknown, the architecture of two closely related T4SSs, one from *Agrobacterium tumefacien*s (VirB/D4 system) and the other from *Escherichia coli* (encoded on the R388 conjugative plasmid), has been examined. The T4SS apparatus is a large macromolecular complex comprising 12 subunits. The complex can be divided into five parts: the stretching needle complex (composed of VirB2), the core/outer membrane complex (composed of VirB7, VirB9, and VirB10), the linking stalk (probably composed of fragments from VirB5 or VirB10), the inner membrane complex (composed of VirB3, VirB4, VirB6, VirB8, and the N-terminus of VirB10), and the ATPases/energy center (consisting of VirB4 andVirB11) (Fronzes et al., 2009; Low et al., 2014; Trokter et al., 2014). Except for VirB1, VirB7, and VirB12, all the other subunits play crucial roles in the virulence of *Brucella* (as inferred from studies involving mice), which may reflect their status in the whole machinery (Comerci et al., 2001; den Hartigh et al., 2004, 2008; Sun et al., 2005).

The underlying molecular mechanisms involved in the initiation of T4SS assembly are still unknown; however, some intracellular signals are thought to be critical for inducing the assembly of T4SS. Several transcriptional regulators may be involved in this process. The low pH condition that occurs within the BCV (as a result of acidification when fusing with the lysosome) probably serves as the key signal that induces the upregulation of the *virB* operon in the bacteria (Porte et al., 1999; Boschiroli et al., 2002; Köhler et al., 2002). Regulators that regulate the expression of the *virB* operon include vacuolar hijacking *Brucella* regulator VjbR (Arocena et al., 2010), *Brucella* luxR-like regulator (BlxR) (Rambow-Larsen et al., 2008), histidine utilization regulator (HutC) (Sieira et al., 2010), the two-component regulatory system BvrR/BvrS (López-Goñi et al., 2002; Martínez-Núñez et al., 2010), RelA/SpoT homolog (Rsh) (Dozot et al., 2006), MarR-like sodium deoxycholate-responsive activator (MdrA) (Sieira et al., 2012), *Brucella* quorum-sensing regulator (BabR) (Caswell et al., 2012), and integration host factor (IHF) (Sieira et al., 2004). However, their exact roles in the induction and assembly of T4SS during infection remain to be evaluated.

### Role of T4SS during infection

Since its identification, T4SS from *Brucella* has been studied extensively. Several *in vitro* and *in vivo* studies have improved our understanding of the phenotypes, including long-term survival within the host, immune inhibition, and the underlying mechanism of action of T4SS. Most of these studies have concentrated on four critical aspects of *Brucella* infection: phenotypes of the infected animals, surviving ability in cultured cells, utilization of the host vesicle trafficking pathway, and regulation of the host inflammatory response. The results indicate that T4SS plays crucial roles in the inhibition of the host innate immune response and in intracellular survival during infection.

In animal models, mainly mice and goats, screening of genes involved in *Brucella* virulence by signature-tagged mutagenesis (STM) led to the identification of T4SS as the crucial factor required to establish successful infection *in vivo* (Hong et al., 2000; Lestrate et al., 2000, 2003; Kahl-McDonagh et al., 2006; Zygmunt et al., 2006). In these studies, screening for genes essential for the acute phase of infection did not identify T4SS; however, screening for genes required for chronic persistence identified T4SS as the virulence determinant (Hong et al., 2000; Lestrate et al., 2003). Consistent with these results, subsequent analysis of the kinetics of infection in mice and calves revealed that T4SS is not needed for initial systematic colonization within the spleen, liver, kidney, and lungs, but is required for chronic persistence (Rajashekara et al., 2005; Rolán and Tsolis, 2007; Rossetti et al., 2013). In goats, a similar screening experiment suggested that T4SS is involved in bacterial survival *in vivo*. Another study reported that, unlike wild-type *B. melitensis* 16M, attenuated *B. melitensis* lacking *virB2* does not cause abortions or colonize fetal tissues in pregnant goats (Kahl-McDonagh et al., 2006; Zygmunt et al., 2006). Recent studies on T4SS in two less virulent strains, *B. ovis* and *B. microti*, revealed that T4SS-deficient strains are significantly attenuated in both the early and late stages of infection (Hanna et al., 2011; Sá et al., 2012).

In cultured cells, including THP1, J774, dendritic cells, murine bone marrow-derived macrophages (BMMs), primary human monocytes, and HeLa cells, T4SS has been shown to be essential for the intracellular growth of *Brucella* during infection (O'Callaghan et al., 1999; Foulongne et al., 2000; Pizarro-Cerdá et al., 2000; Sieira et al., 2000; Comerci et al., 2001; Delrue et al., 2001; Celli et al., 2003; Salcedo et al., 2008; den Hartigh et al., 2008). Screening for the intracellular growth ability of *Brucella* mutants in HeLa and THP1 cells revealed that the disruption of T4SS leads to impaired intracellular survival, thereby demonstrating the importance of this secretion machinery *in vitro* (Foulongne et al., 2000; Delrue et al., 2001). Among the proteins that comprise the T4SS apparatus, VirB2–6 and VirB8-11, were found to be essential for the survival and replication of *Brucella* within host cells (Guzman-Verri et al., 2002; Celli et al., 2003; Fronzes et al., 2009; Lee et al., 2013; Trokter et al., 2014), indicating the importance of the integrity of this secretion apparatus and the differing roles of its various components. T4SS-deficient mutants exhibited complete loss of the ability to grow within cells, *in vitro*; however, they exhibited no attenuation in the initial systematic spreading stage, albeit a diminished replicating ability during the later stage was observed *in vivo*. This suggests that, in the initial invasion stage of an *in vivo* infection, bacterial survival does not rely on T4SS. This may be attributed to the extracellular localization of the bacteria and the favorable microenvironment. These aspects need to be studied in more detail in the future.

To elucidate the mechanism by which T4SS mediates the intracellular survival ability of *Brucella*, VirB10 polar mutants, which lacked the *virB10* gene and were therefore, deficient in T4SS, were used. The results revealed that T4SS-deficient *Brucella* still invaded host cells and interacted with the early and late endosomes in a manner similar to that by wild-type bacteria, as suggested by the acquisition of the early and late endosome markers (EEA1 and LAMP1, respectively) by the BCVs (Delrue et al., 2001; Celli et al., 2003). However, unlike wild-type *Brucella*, BCVs of the mutant bacteria did not exclude LAMP1 over time, and remained LAMP1-positive. Therefore, these mutants were engulfed by lysosomes and eventually targeted for degradation (Comerci et al., 2001; Delrue et al., 2001; Celli et al., 2003). Thus, unlike wild-type *Brucella*, the T4SS-deficient mutants did not complete their intracellular lifecycle, which involves escaping from the late endosome, reaching the ER and establishing the replicating niche, and escaping from the ER to utilize autophagic vacuoles for survival (Starr et al., 2012). Therefore, what happens to the BCVs within the late endosome/lysosome is very critical, and determines the fate of intracellular *Brucella*. This divergence may depend on the induction of the *virB* operon and subsequent delivery of the effectors into the host cytosol. The signals that trigger the induction may originate from the environment around *Brucella*, such as acidification during BCV fusion with the late endosome/lysosome (Pizarro-Cerdá et al., 1998; Starr et al., 2008).

*Brucella* use the stealth strategy to avoid immune recognition by the host, and elicit a much lower immune response compared to other bacterial infections, such as those caused by *Salmonella* (Barquero-Calvo et al., 2007). Although T4SS may play a role in regulating the host immune response, the molecular mechanism underlying this regulation is unclear. Some studies have indicated that T4SS is required for *Brucella* to elicit relatively low levels of immune responses (Rolán and Tsolis, 2007, 2008; Rolán et al., 2009). Conversely, experiments performed on other bacteria have indicated roles for T4SS in inhibiting host immune responses, as evidenced by the fact that T4SS-deficient mutants generally induce a stronger immune response in the hosts than wild-type strains (Luo, 2012; van Schaik et al., 2013). The phenotype of the infected cells suggested that T4SS-deficient *Brucella* are quickly degraded in the lysosomes, before they can ignite an immune response. On the other hand, wild-type *Brucella* activate the host immune response at a later stage in their intracellular lifecycle. Another possibility is that the effectors secreted by T4SS have inflammation-inhibitory activities within host cells. Therefore, we believe that the loss of a functional T4SS may lead to impaired effector secretion and upregulated immune responses.

Like for many other bacteria, T4SS is essential for the virulence of *Brucella* infections, in both animal models and cultured cells. The cellular and molecular mechanisms underlying this virulence may include hijacking the vesicle trafficking pathway and manipulating the innate and adaptive immune responses of the host. T4SS secretes effectors into host cells. These effectors then target various host mechanisms and perpetuate the infection. Therefore, identifying the effectors secreted by *Brucella* through the T4SS machinery and determining their target host pathways are critical for understanding their pathogenesis.

### Molecular events that are potentially targeted by *Brucella* T4SS effectors

**Figure 1:**
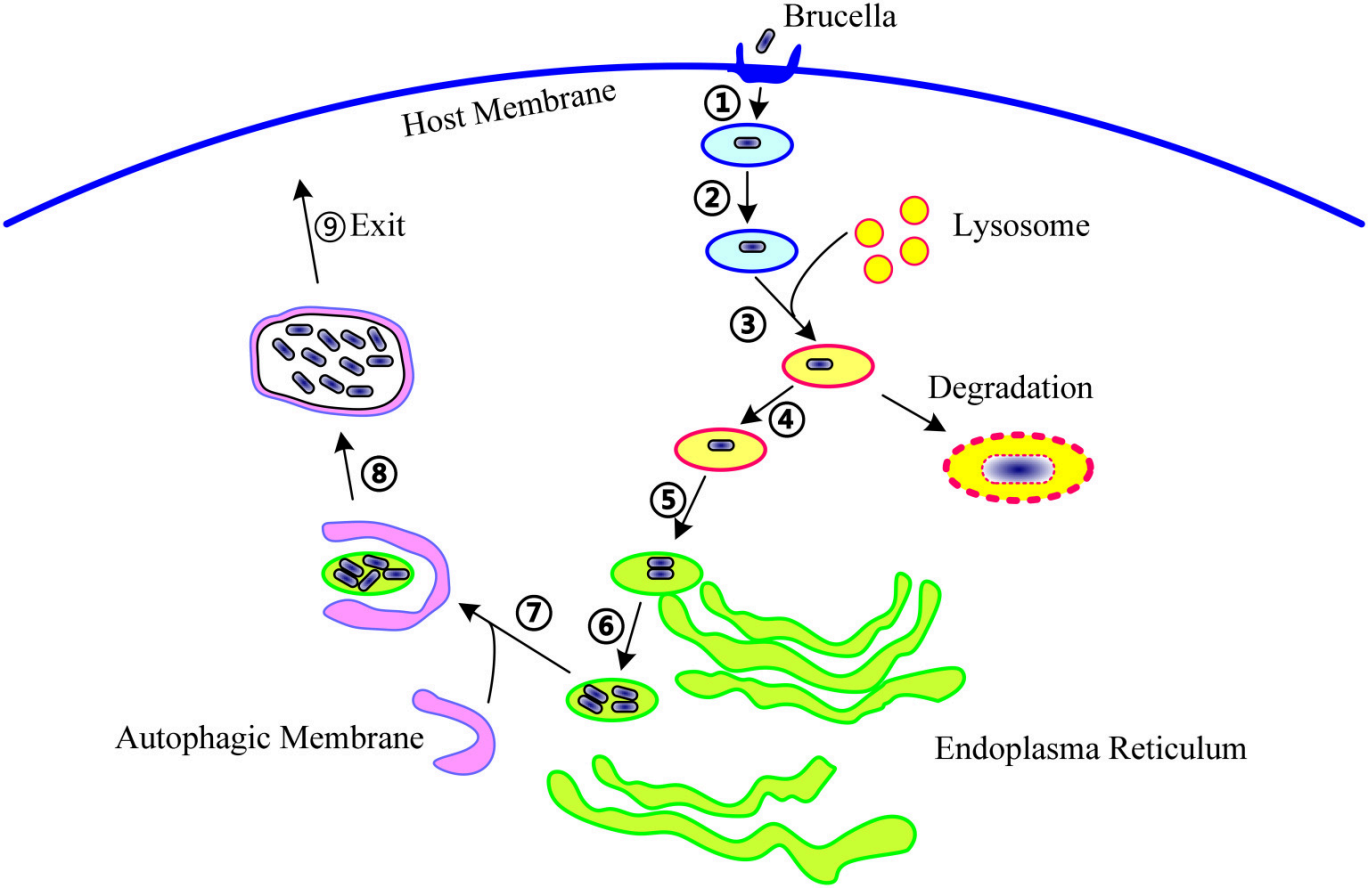
**The life cycle of ***Brucella*** within host cells**. During infection, *Brucella* first invade the host cells (①), form *Brucella*-containing vacuoles (BCVs) (②), and undergo fusion with the lysosome in a controlled manner (③). In this step, about 90% of the *Brucella* are degraded, and the remaining 10% survive (④). Then, the BCV traffic to and reach the endoplasmic reticulum (ER) (⑤), and establish the replicative site (⑥). After ER replication, the *Brucella* traffic toward the autophagy-like vacuoles (⑦), survive within these compartments (⑧), and finally, leave the host cells to promote cell-to-cell spreading (⑨). The following molecular events involved in these steps are potentially targeted by T4SS: excluding markers of the late endosome or lysosome, acquiring ER markers, interacting with secretory pathways, acquiring markers for autophagosomes, resisting the harsh intracellular environment, and regulating the activation of vital immune pathways.

To date, 15 T4SS effectors have been identified in *Brucella*. The phenotypes presented by T4SS-deficient mutants and studies on some well-known T4SS effectors from other bacteria suggest that, in *Brucella*, T4SS effectors might function in the following molecular events associated with infection: (1) excluding markers of late endosome or lysosome, (2) acquiring ER markers, (3) interacting with secretory pathways, (4) acquiring markers for autophagosomes, (5) resisting the harsh intracellular environment, and (6) regulating the activation of vital immune pathways (Figure 1). However, their exact mechanisms of action have not been clarified. Every event may be targeted by one or more effectors, and an effector may act on one or more processes. In addition, the effectors may exhibit overlapping functions, thereby complicating the elucidation of their individual functions.

## Known effectors secreted by *Brucella* through T4SS

### Identification of effectors

Although T4SS was identified in *Brucella* spp. approximately 15 years ago, the effectors of T4SS remained uncharacterized until recently. The first two effectors, VirB-co-regulated effector (Vec) A and VecC, were identified while screening for genes whose transcription was co-regulated by the *virB* operon regulator, VjbR (de Jong et al., 2008). Screening of the interactions between human proteins and predicted *Brucella* proteins using a yeast two hybrid (Y2H) system led to the identification of the Rab2 interacting conserved protein A (RicA)-Rab2 interaction pair. Subsequently, RicA was identified as a T4SS-dependent effector (de Barsy et al., 2011). Genome-wide bioinformatics screening for putative T4SS effector proteins using distinct filtering criteria led to the identification of nine T4SS-dependent effector proteins (Marchesini et al., 2011; Myeni et al., 2013). Bacterial toll-Interleukin receptor (TIR) domain-containing proteins are thought to be involved in the virulence of *Brucella*; screening for TIR-containing proteins in *Brucella* led to the identification of two proteins, *Brucella* TIR protein (Btp) A and BtpB, as effectors that are translocated by T4SS into host cells (Salcedo et al., 2013). Recently, a secretory protein, secreted effector protein A (SepA), which is encoded within a horizontally transmitted region, was confirmed as a novel T4SS substrate (Döhmer et al., 2014). Together, these 15 effectors constitute the repertoire of T4SS substrates in *Brucella* spp. identified to date (Table 1).

**Table 1:** **The 15 effectors secreted by ***Brucella*** through T4SS**.

**Name**	**ORF in *B. abortus***	**Amino acids**	**Conserved domains**	**Targets within hosts**	**Functions**	**Methods**	**References**
RicA	BAB1_1279	175	Carbonic anhydrase	Rab2	Regulating vesicle trafficking	TEM1	de Barsy et al., 2011; Nkengfac et al., 2012; Herrou and Crosson, 2013
VceA	BAB1_1652	105	Non	Unknown	Unknown	CyaA, TEM1	de Jong et al., 2008
VceC	BAB1_1058	418	The Proline-rich domain	Bip/Grp78	Activating Unfolded Protein Response	CyaA, TEM1	de Jong et al., 2008, 2013
BPE005	BAB1_2005	153	The Coiled-coil domain	Unknown	Unknown	CyaA	Marchesini et al., 2011
BPE043	BAB1_1043	1553	The Cyclic nucleotide binding domain	Unknown	Unknown	CyaA	Marchesini et al., 2011
BPE275	BAB1_1275	253	The Rhomboid-like domain	Unknown	Unknown	CyaA	Marchesini et al., 2011
BPE123	BAB2_0123	153	The Apolipoprotein domain	Unknown	Unknown	CyaA	Marchesini et al., 2011
BtpA/TcpB/Btp1	BAB1_0279	250	TIR domain	MAL	Inhibiting TLR pathways	CyaA	Cirl et al., 2008; Radhakrishnan et al., 2009; Radhakrishnan and Splitter, 2010; Sengupta et al., 2010; Salcedo et al., 2013
BtpB	BAB1_0756	292	TIR domain	Unknown	Inhibiting TLR pathways	CyaA, TEM1	Salcedo et al., 2013
BspA	BAB1_0678	191	The Pfam domain DUF2062	Unknown	Inhibiting the secretory pathway	CyaA, TEM1	Myeni et al., 2013
BspB	BAB1_0712	187	The SCOP structural domain (d2gsaa)	Unknown	Inhibiting the secretory pathway	CyaA, TEM1	Myeni et al., 2013
BspC	BAB1_0847	137	The predicted Sec-dependent signal peptide	Unknown	Unknown	CyaA, TEM1	Myeni et al., 2013
BspE	BAB1_1671	117	Coiled-coil (CC) and TM domains	Unknown	Unknown	CyaA, TEM1	Myeni et al., 2013
BspF	BAB1_1948	428	the GNAT-family acetyltransferase domain	Unknown	Inhibiting the secretory pathway	CyaA, TEM1	Myeni et al., 2013
SepA	BAB1_1492	130	Non	Unknown	Inhibiting BCV fusion with the lysosome	TEM1	Döhmer et al., 2014

A candidate protein may be identified as a T4SS effector protein if it fulfills two criteria: the protein must be secreted into host cells and the secretion must be through the T4SS machinery. The former may be validated by TEM-1 lactamase or calmodulin-dependent adenylate cyclase (CyaA) assays and the latter, by constructing a T4SS-deficient mutant. All strategies used to identify effectors in *Brucella* are confined by the limited number of potential proteins; similar problems were encountered during the identification of effectors in other bacteria as well. By extending the screening of potential targets to the whole genome, the Dot/Icm T4SS machinery of *Legionella pneumophila* and *Coxiella burnetii* were identified to secrete approximately 300 and 100 proteins, respectively (Chen et al., 2010; Voth et al., 2011; Zhu et al., 2011; Lifshitz et al., 2013). Similarly, extending the screening method described above to the whole *Brucella* genome may lead to the identification of many more effectors.

### VceA and VceC

Both VceA and VceC, which are composed of 105 and 418 amino acids, respectively, are highly conserved in all *Brucella* spp. They share an 18-base pair (bp) conserved promoter box, similar to the *virB* promoter, and their transcription is regulated by the same transcriptional activator, VjbR (de Jong et al., 2008). Like in the case of the *virB* promoter, VjbR expressed under *in vitro* conditions could bind to DNA fragments containing the VceC promoter in electrophoretic mobility shift assays (EMSA). Expression of v*ceC* and *virB* was downregulated in *B. abortus vjbR* mutants, as determined by lacZ fusion and β-galactosidase activity assays (de Jong et al., 2008), suggesting that both genes are co-regulated by the same transcriptional factor.

Analysis of the various domains of VceC revealed that 20 amino acids present at its C-terminus are necessary for its translocation into host cells, and 38 amino acids present at its N-terminus, which form a hydrophobic transmembrane domain, are required for its localization to and perturbation of the ER (de Jong et al., 2008, 2013). Immunoprecipitation-mass spectrometry (IP-MS) studies revealed that the ER chaperone protein, Bip/Grp78, binds to the central proline-rich region of VceC. This proline-rich region is the only characterized domain of VceC (de Jong et al., 2013). Consistent with the localization and identity of its interacting partners inside host cells, VceC was shown to activate the unfolded protein response (UPS), cause ER stress, and induce pro-inflammatory responses during infection. The N-terminal ER-targeting domain of VceC was found to be involved in the activation of these processes (de Jong et al., 2013). However, whether the activation of UPS by VceC contributes to its cytotoxity toward macrophages is not clear (de Jong et al., 2008). Further studies are required to elucidate the nature and consequences of the interaction between VceC and Bip. In addition, the co-regulation of *vceC* and *virB* by VjbR suggests that the expression of *virB* and its effectors might be temporally regulated in response to the microenvironment around the BCVs.

### RicA

RicA is a mono-domain protein composed of 175 amino acids (de Barsy et al., 2011). Extensive mutagenesis studies have revealed that there are two key elements in RicA, a β-sheet and an isoleucine-glycine-phenylalanine-proline (IGFP) loop, both of which are thought to be involved in protein folding and interaction with Rab2 (Nkengfac et al., 2012). The crystal structure of RicA revealed that the structure of this protein is highly similar to those of the γ-carbonic anhydrase (CA) family of proteins, all of which contain a bound zinc ion. However, RicA does not possess carbonic anhydrase activity, suggesting that it may have evolved a unique function that remains to be elucidated (Herrou and Crosson, 2013).

RicA interacts both *in vivo* and *in vitro* with guanosine diphosphate (GDP)-free and GDP-bound forms of Rab2, but not with the GTP-bound form, suggesting that RicA is not a GTPase-activating protein (GAP) (de Barsy et al., 2011; Herrou and Crosson, 2013). Further assays revealed that this protein does not function as a guanine nucleotide exchange factor for Rab2. Whether RicA functions as a GDP dissociation inhibitor or a Rab escort protein remains to be clarified. A *ricA* deletion mutant did not exhibit attenuated virulence in either mice or HeLa cells. In addition, the internalization and intracellular survival abilities of the mutant remained unaltered (de Barsy et al., 2011). Surprisingly, Δ*ricA* mutants showed faster loss of LAMP1 inside host cells compared with wild-type *Brucella* (de Barsy et al., 2011), suggesting that Δ*ricA* mutants escape earlier from late lysosomes, arrive at the ER, and establish a replicative niche faster than wild-type bacteria.

### BtpA and BtpB

BtpA (also known as Btp1/TcpB) and BtpB share a conserved TIR-containing domain, which is widely present in mammals and is responsible for mediating the signaling cascades of innate immune recognition (Salcedo et al., 2013). Both these proteins were confirmed to be translocated into host cells, as verified by the CyaA assay; however, in the widely used TEM-1 lactamase assay, BtpB showed markedly lower translocating efficiency than did other effectors or BtpA. However, one must note that the TEM-1 lactamase assay is less sensitive than the CyaA assay (Salcedo et al., 2013). Both proteins inhibit the maturation of dendritic cells and are essential for virulence; however, they show opposite effects on the activation of NF-κB, with BtpA inhibiting and BtpB inducing the activation of NF-κB (Salcedo et al., 2008, 2013; Sengupta et al., 2010). The function and mechanism of action of BtpA has been studied extensively (Cirl et al., 2008; Radhakrishnan et al., 2009; Radhakrishnan and Splitter, 2010; Sengupta et al., 2010), and a recent crystal structure revealed that BtpA specifically targets the host signaling adapter protein, MyD88 adapter-like (MAL)/TIRAP, through a unique BB loop region within the TIR domain (Alaidarous et al., 2014; Snyder et al., 2014).

In order to mediate the inhibition of the host TLR signaling pathway, BtpA must be translocated into the host cell from the bacteria; for *Brucella*, the T4SS machinery is probably the most efficient system for exerting this translocation. TIR domain-containing proteins are present in many other bacteria, such as TlpA in *Salmonella enterica*, TcpC in *Escherichia coli* strain CFT073, TirS in *Staphylococcus aureus*, YpTdp in *Yersinia pestis*, and PdTLP in *Paracoccus denitrificans*. These proteins also have the ability to inhibit the toll-like receptor (TLR) pathway (Newman et al., 2006; Chan et al., 2009; Spear et al., 2009; Rana et al., 2011, 2013; Snyder et al., 2013; Askarian et al., 2014); whether or how they are delivered into host cells during infection remains to be elucidated.

### BspA, BspB, and BspF

BspA, BspB, and BspF, together with BspC and BspE, were first predicted by bioinformatics approaches and then identified by the TEM-1-fusing protein method (Myeni et al., 2013). BspA, BspB, and BspF are composed of 191, 187, and 428 amino acids, and contain the DUF2062 domain (Domain of Unknown Function 2062, Pfam database), the structural classification of proteins (SCOP) structural domain (flanked by 2 transmembrane domains), and the Gcn5-related N-acetyltransferases (GNAT)-family acetyltransferase domain, respectively (Myeni et al., 2013). Ectopically expressed BspA and BspB appear to localize at the ER, while BspF localizes throughout the cytosol and plasma membrane (Myeni et al., 2013). These three effectors inhibit the host cells' protein secretory pathway when overexpressed in transfected cells, and inhibit cellular secretion during infection (Myeni et al., 2013). Although deletion of one of these effectors alone did not impair the replicating abilities of the mutants in infected BMMs, as determined by enumerating colony forming units (CFUs), the triple Δ*bspABF* mutant showed decreased intracellular growth (Myeni et al., 2013). Nevertheless, both Δ*bspB* and Δ*bspABF* mutants displayed a similar replication deficiency when the percentage of infected cells supporting bacterial replication and containing at least 10 bacteria were scored (Myeni et al., 2013). Consistent with these results, in mice models, the triple Δ*bspABF* mutant partially lost its ability to survive in the liver, in contrast to the wild-type strain and the single deletion mutants (Myeni et al., 2013). However, neither single nor triple deletions of these three effectors affected the ability to establish systematic infection (Myeni et al., 2013), suggesting that these effectors exert their effects only in the later stages of infection and have complementary functions. Further research on these effectors needs to focus on the identification of their interacting partners, elucidation of their biochemical mechanisms, and resolution of their crystal structures.

### SepA

SepA is a 130-amino acid protein that does not contain any previously characterized domain. Secretion of SepA occurs in the very early stages of infection (as early as 30 min post-infection), in a T4SS-dependent manner (Döhmer et al., 2014). However, strikingly, SepA is not directly delivered into host cells. Instead it goes through a periplasm-aggregating intermediate stage before leaving the bacterium, unlike other effectors secreted by bacterial T4SS or T3SS (Döhmer et al., 2014). A Δ*sepA* mutant exhibited slightly diminished replicating ability 48 h post-infection in infected macrophages, and dramatically decreased replicating ability in the first 4–24 h post-infection, suggesting that SepA is involved in the initial stages of intracellular survival, consistent with its early secretion (Döhmer et al., 2014). The absence of attenuation in the Δ*sepA* mutant might be because other effectors have similar functions. As the intracellular cycle of *Brucella* proceeds, the effects of SepA deficiency may be rescued by these effectors, resulting in attenuation at the initial stages and recovery at later stages. Interestingly, although the Δ*sepA* mutant was as virulent as the wild-type strain, a SepA-overexpressing mutant was found to be attenuated, indicating that the expression of SepA is tightly regulated (Döhmer et al., 2014). Because the other effectors exert their effects in the later stages of the intracellular lifecycle of *Brucella*, early secretion is a unique feature of SepA. Therefore, *sepA* mutants may be used to study the early stages of infection by *Brucella*.

### Other effectors

BspC, BspE, BPE123, BPE005, BPE275, and BPE043 were identified as effectors after experimental validation of potential proteins as described above. Although they are highly conserved among the *Brucella*, little is known about their roles and mechanisms of action. When expressed ectopically, both BspC and BspE localize around the cell nucleus, but with some differences; the former possibly co-localizes with the Golgi apparatus but the latter forms discrete vesicles (Myeni et al., 2013). BspC contains an N-terminal Sec-dependent signal peptide and can induce an ER stress response without affecting the protein secretion pathway (Myeni et al., 2013). BPE005, BPE275, and BPE043 are widely found in other kinds of bacteria, and BPE123 is present in several species, including *Bartonella bacilliformis, Ochrobactrum anthropi*, and *O. intermedium* (Marchesini et al., 2011). BPE123 is positioned at the surface of the BCV during infection. Twenty-five amino acids located at the N-terminus of the protein might function as a signal sequence for its translocation into the host cell (Marchesini et al., 2011). The multiplication abilities of BPE123-deficient and wild-type strains were found to be indistinguishable in several cell types and mouse models (Marchesini et al., 2011).

## Future perspective

The identification of the effectors secreted by *Brucella* will enable the elucidation of the functions and mechanism of action of T4SS. In addition, it facilitates the comparison of the various roles of T4SS among gram-negative bacteria. Effectors secreted by T4SS from different bacteria, albeit with similar sequences, may display distinct structures, functions, and mechanisms, contributing to diverse bacterial pathogenesis. Because *Brucella* is used as a model organism to study intracellular infections, studying the effectors from this species might provide insights into the functioning of T4SS in intracellular bacteria, which may, in turn, aid in understanding the pathogenesis of intracellular bacterial pathogen.

Compared to other bacteria, studies on *Brucella* T4SS are limited in number, partly because the effectors were identified only recently. On the basis of the information available on the pathogenesis of *Brucella*, we believe that the following aspects should be considered in future studies: (1) characterization of the biochemistry of these effectors, including identification of the binding targets within host cells, elucidation of the biochemical mechanisms underlying their action on targets, investigations on whether they contain featured domains or novel enzymatic activities such as kinase or phosphatase activities etc., and their intracellular co-localization with binding partners when ectopically expressed or injected during infection; (2) characterization of the cell biology of these effectors, including their effects on intracellular persistence, intracellular apparatus or cytoplasmic membrane docking, signaling pathways targeted by them, and regulation of BCV trafficking [including evading fusion with lysosomes, arriving at ERs, moving to autophagic compartments, and spreading into neighboring cells (see Figure 1)]; (3) elucidation of the contribution of these effectors to virulence *in vivo*, including their necessity, roles in establishing systematic infection, processing during chronic infection, or eliciting inflammatory response, and whether these effectors can explain the typical intracellular survival performance of *Brucella* species; (4) investigation of effector microevolution, including similarities and diversities among closely associated species, distribution of homologs in other intracellular bacterial pathogens, horizontal transfer among bacteria, and conservation of function or structure with proteins in other bacteria.

In past decades, our knowledge of vacuolar trafficking of eukaryotic cells has significantly advanced; as a result, cues and tools to explore the intracellular life style of *Brucella* spp. are now available. Here, on the basis of the information available on the pathogenesis of *Brucella* and the roles of its T4SS outlined above and elsewhere (Atluri et al., 2011; Martirosyan et al., 2011; Bargen et al., 2012), we suggest that the following events are targeted by the 15 effectors of *Brucella* during infection (Figure 1): acquiring or losing markers for late endosomes, ERs, or autophagosomes; resisting and surviving the harsh microenvironment; hijacking and utilizing the secretory pathways of the ER and the Golgi; and usurping or regulating the innate immune response. Analyzing the roles of all 15 effectors in these cascades will further our understanding of *Brucella* pathogenesis.

Effectors that are translocated into host cells by bacteria provide an interface between the bacterium and the host, and lie in the frontline of the battle between the two. Studying the effectors is not only important for understanding bacterial pathogenesis but also provides insights into the immune response of eukaryotic hosts. The lack of an adequate immune response is one of the primary reasons for the inability of the host to overcome the *Brucella* infection (Martirosyan et al., 2011; Bargen et al., 2012). Therefore, studying the effectors secreted by *Brucella* might provide insights into the mechanisms by which this intracellular bacterium specifically hijacks the host immune signaling pathways. Knowledge of these mechanisms will help develop better vaccines and therapies against *Brucella*.

### Conflict of interest statement

The authors declare that the research was conducted in the absence of any commercial or financial relationships that could be construed as a potential conflict of interest.
